# Overview of Earth Observation Satellite Platform Microvibration Detection Methods

**DOI:** 10.3390/s20030736

**Published:** 2020-01-29

**Authors:** Xinming Tang, Junfeng Xie, Hong Zhu, Fan Mo

**Affiliations:** 1 Land Satellite Remote Sensing Application Center, MNR, Beijing 100048, China; txm@lasac.cn (X.T.); mof@lasac.cn (F.M.); 2 Key Laboratory of Satellite Surveying and Mapping Technology and Application, NASG, Beijing 10048, China; 3 School of Earth Science and Engineering, Hohai University, Nanjing 211100, China; 4 School of Surveying and Geographical Science, Liaoning Technical University, Fuxin 123000, China; 5 College of Ecology and Environment, Institute of Disaster Prevention, Langfang 065201, China

**Keywords:** satellite platform, microvibration detection, microvibration frequency, geometric positioning accuracy, surveying and mapping application

## Abstract

Satellite platform microvibration is a common phenomenon in earth observation satellite orbits that directly affects the imaging quality and accuracy of surveying and mapping. With the continuous improvement in the spatial resolution, the influence of satellite platform microvibration on image geometric accuracy is becoming increasingly significant. High-precision microvibration detection and compensation are key technologies for eliminating image distortion and location deviation caused by satellite platform microvibration. In this paper, the microvibration detection methods of different satellite platforms are summarized, and the verification and analysis are performed on the data downloaded from the Resource-3 satellite (ZY-3) platform, to provide technical support for subsequent refined processing of satellite attitude.

## 1. Introduction

With the development of satellite sensors, the spatial, temporal, and spectral resolutions of remote sensing data are increasing. Accordingly, higher requirements on the pointing accuracy and attitude stability of the satellite platform itself are being put forward to meet user requirements for high imaging quality and geometric positioning accuracy [1]. Platform microvibration is a common phenomenon when a satellite is in orbit [2,3]. It is caused by the high-speed rotation of rotating parts, mechanical movement of thermal control parts, vibration of large, flexible structures, and changes in the thermal environment. Satellite platform microvibration is also a common technical challenge in the field of remote sensing and satellite data processing at home and abroad [4,5,6]. However, high-frequency microvibrations mainly affect the image quality, while low-frequency microvibrations primarily affect the image geometric positioning accuracy [7,8]. The different frequency microvibrations have adverse effects on the application of remote sensing data. In recent years, experts and scholars at home and abroad have carried out much research on satellite platform microvibration detection methods. Detection tests have been carried out on a number of earth observation satellites, the results of which regarding the vibration frequency and amplitude are shown in Table 1.

Combining the research of domestic and foreign scholars on microvibration analysis with the processing methods for earth observation satellite platforms, when the orbital height and spatial resolution continue to improve, the satellite platform microvibration becomes a key technical problem that restricts the high-precision geometric processing and application of high-resolution optical remote sensing satellites. From the perspective of imaging quality, with the panchromatic image resolution reaching the submeter level, the internal geometric accuracy with consideration of the satellite platform microvibration must be controlled to within one pixel. Assuming that the stability of the satellite platform can reach 1/100 pixel and the corresponding attitude angle is 0.002″, if the attitude corresponding to a pixel error is greater than 0.2″, the optical image needs to be compensated for by the microvibration geometry to improve the image quality. From the perspective of mapping accuracy, the 1:10,000 mapping requires a plane accuracy greater than 5 m and an elevation accuracy better than 1.5 m. The 1:500 mapping accuracy requires a plane accuracy higher than 2.5 m and an elevation accuracy better than 1.2 m. When the geometric error caused by microvibration cannot meet the mapping accuracy, the geometric positioning accuracy needs to be improved through attitude compensation. The impact of platform microvibration on satellite image mapping at the submeter or even higher level of resolution in the future cannot be ignored [27,28]. As the agility of the satellite platform improves, the impact of microvibration on the geometric accuracy of satellite images will become more significant. Therefore, the microvibration detection methods and characteristics of the satellite platform are summarized and analyzed in this paper. Resource-3 satellite (ZY-3) is the first civil three-dimensional mapping satellite and transmitted a lot of original data in China. Experimental verification with the attitude, image, and other data transmitted by the ZY-3 satellite is carried out to provide theoretical and technical support for the detection of microvibrations on the domestic remote sensing earth observation satellite.

## 2. Satellite Platform Microvibration Detection Method

Many methods for detecting satellite platform microvibration at home and abroad exist. According to the reference object, satellite platform microvibration methods can be classified into two categories: direct detection methods based on an attitude sensor and indirect detection methods based on a non-attitude sensor. The two methods are summarized and compared in the following.

### 2.1. Direct Detection Method Based on an Attitude Sensor

**Figure 1:**
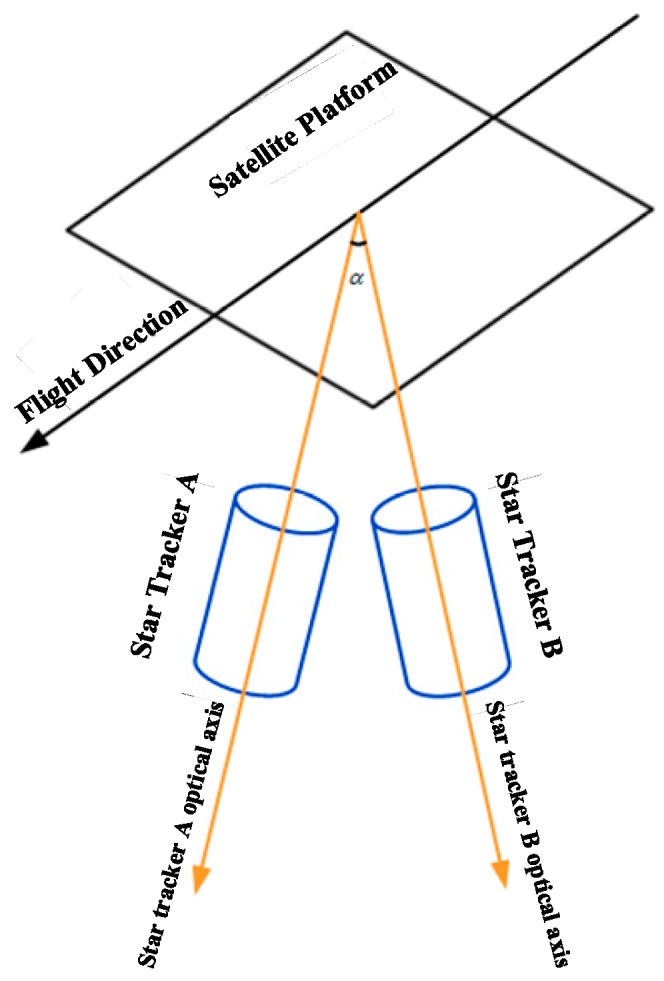
Star tracker schematic diagram.

The direct detection method based on an attitude sensor mainly uses the attitude angle or the angular velocity measurement output to carry out direct detection. This kind of detection method includes the use of an attitude measurement load, such as a star sensor, a gyroscope, or an angular displacement tracker (ADS). The details are as follows: (1) Microvibration detection based on the attitude of the star sensor. The attitude quaternion output by the star sensor is taken as a reference [29]. The attitude variation is obtained by differential attitude data, and the frequency and amplitude of the platform vibration are obtained by spectral analysis. Mo et al. [30], based on the satellite-sensitive attitude data of the ZY-3 satellite, obtained the microvibration of the platform, with a frequency range of 0.64~0.71 Hz. (2) Microvibration detection based on the angle of the optical axis of the multistar sensor. As shown in Figure 1, the difference between the measured and installed values of the included angle of the star-sensitive optical axis and the change in the included angle of the optical axis with time were obtained [31], and the Fourier transform was used in spectrum analysis to obtain the frequency and amplitude of the platform microvibration. Tang et al. [32] detected the microvibration of a platform with a frequency of approximately 0.0011 Hz using the included angle of the star-sensitive optical axis. (3) Microvibration detection based on an angular velocity sensor. Angular velocity sensors such as gyros and ADSs can directly output attitude angular velocity data at a higher frequency [33,34,35], and the amplitude and frequency of platform microvibration can be directly obtained by Fourier spectrum analysis. The microvibration frequency range of the ETS-VI satellite detected by Toyoshima [36] was 0.39~250 Hz, of which 83.6% was concentrated in 0.39~10 Hz and over 99% was below 102 Hz. Ran et al. [15] detected a 200 Hz microvibration in the frequency domain for the Beijing-1 satellite platform.

### 2.2. Indirect Detection Method Based on a Non-Attitude Sensor

**Figure 2:**
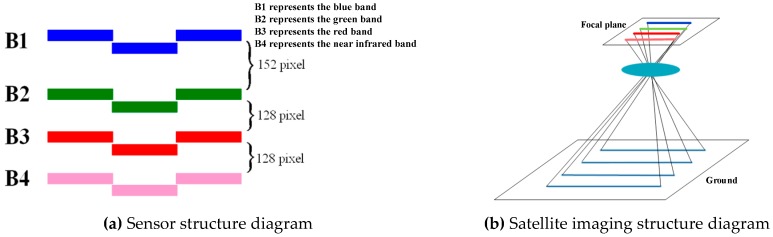
Schematic diagram of microvibration detection based on multispectral images [30].

**Figure 3:**
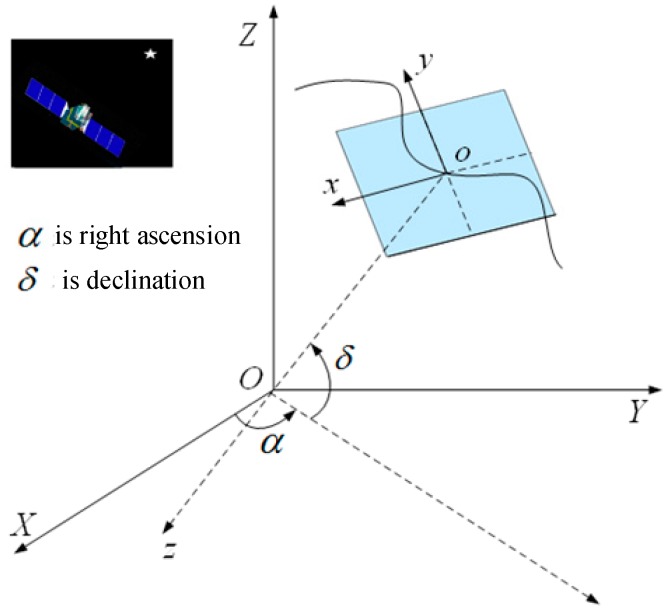
Diagram of satellite microvibration detection based on star map.

**Figure 4:**
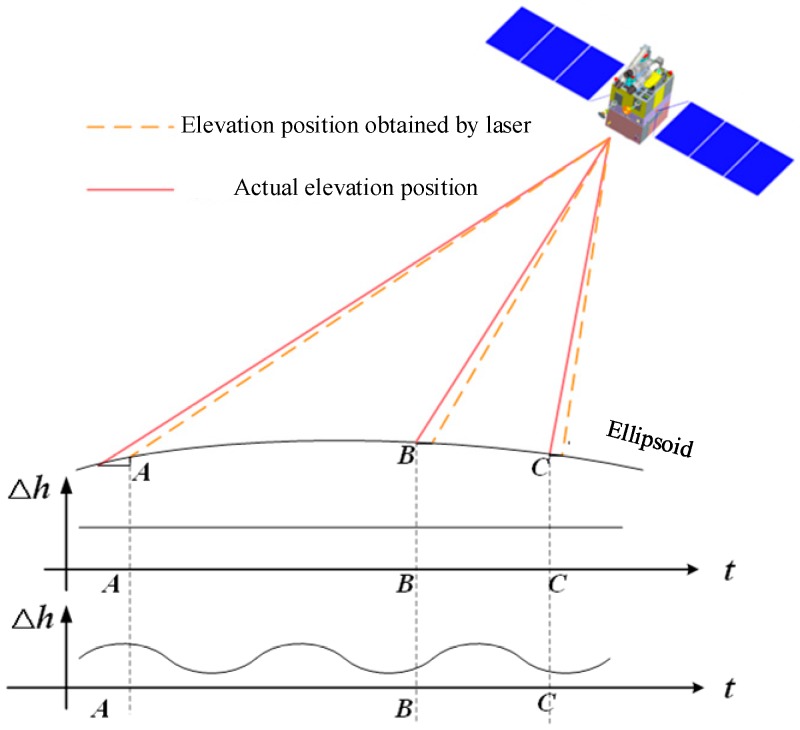
Schematic diagram of satellite platform microvibration detection based on laser altimeter.

**Figure 5:**
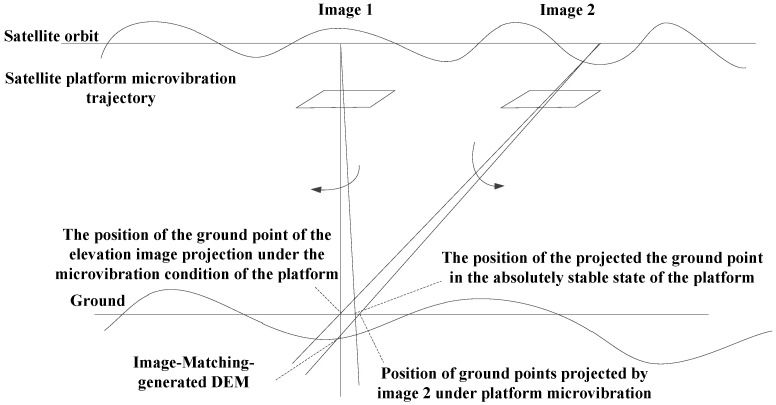
Diagram of microvibration detection based on surveying and mapping products [14].

The indirect detection method based on the non-attitude sensor mainly relies on other load data on the platform, such as the output data of the ground camera, star camera, laser altimeter, etc., to detect the platform microvibration, as follows: 

(1) Microvibration detection based on satellite images. The satellite image matching parallax detection method is typically used to carry this out. As shown in Figure 2, this category mainly includes microvibration detection methods based on multispectral images and multi-non-collinear TDI CCD images [37]. John [11] and Akira et al. [38] proposed using the parallax between parallel linear array CCDs to detect the microvibrations of satellite platforms [25,32,33,34]. Ayoub et al. [10] used image registration to detect the microvibrations of the SPOT5 satellite platform and detected microvibrations with a frequency of approximately 0.003 Hz. Tong [21], Wang [26], and Sun et al. [39] used multispectral images to detect the microvibration frequencies of the ZY-3 satellite platform around 0.65 Hz. Jiang et al. [40] used multispectral images to detect and compensate for the microvibrations of the ZY-1 02C satellite platform.

(2) Microvibration detection based on the star map. Amberg [20] and Mo [30] successively proposed a microvibration detection method based on a star map that takes the stars as the reference, indirectly detecting satellite platform microvibrations through the offset of the star spot mass center and the star image point fitting track, as shown in Figure 3. Mo [30] detected a microvibration of approximately 0.59 Hz on the satellite platform by using the map image of the ZY-3 satellite.

(3) Microvibration detection based on a laser. This procedure is mainly divided into two methods: microvibration detection based on laser footprint coordinates and microvibration detection based on laser footprint image points. The former detects the satellite platform microvibration by analyzing the regularity of the difference between the elevation obtained by the laser altimeter and the actual elevation on the ground [41,42], as shown in Figure 4. The latter indirectly detects the satellite platform microvibration through the offset of the fitting track of the center of mass and the center of mass of the footprint image; the process is similar to that of star map detection. Tang and others [43,44] found that the platform has microvibrations by using the laser footprint coordinate data of ZY3-02.

(4) Microvibration detection based on surveying and mapping products. This method involves using satellite image and ground control data (DEM and DOM products) to match points of the same name and detecting satellite platform microvibrations [16,44] through image square residuals, as shown in Figure 5. Takaku [16] and Tong et al. [44] demonstrated the specific theory and carried out relevant experimental verification. Gwinner [14] and others used the DEM data generated as ground control data to detect the microvibration frequency range of a satellite platform, i.e., from 0.1~0.2 to 1.7 Hz.

### 2.3. Comparative Analysis of Microvibration Detection Methods

Whether the satellite platform microvibration can be detected requires the following two necessary conditions: first, the frequency range of the platform microvibrations should be within the limited range of the detection reference data frequencies. Second, the detection sensitivity of the reference data should be higher than the amplitude of the platform microvibrations. In addition, due to the influence of reference data acquisition, accuracy, and other factors, the universality of different methods should not be the same. Therefore, this paper analyzes the two types of detection methods relative to the detection frequency range, detection sensitivity, universality, etc., as shown in Table 2, Table 3 and Table 4.

(1) In terms of the detection frequency range, Table 1 reveals that the sampling frequency of the attitude tracker is the key factor determining the detection frequency of the platform microvibration. According to Shannon Theory, the theoretical limit frequency for detecting microvibrations is less than half the output frequency of the attitude tracker. These methods mainly include the absolute attitude and relative attitude. Since the measurement target is different, to obtain the absolute attitude at a single moment, absolute attitude trackers such as star trackers and earth infrared trackers tend to have low detection frequencies, typically dozens of hertz. Trackers that measure the relative angular velocity, such as ADSs, can measure frequencies up to hundreds of hertz, and the spectrum of the satellite platform microvibration is greatly increased.

(2) In terms of detection sensitivity, Table 2 shows that the accuracy of the detection method based on the pose sensor depends on the measurement accuracy of the pose sensor equipment itself. This kind of method mainly uses the angular displacement and angular velocity to record the pose information of the satellite platform or combines the satellite sensor and gyro to obtain the absolute pose information of the satellite platform. Notably, the platform microvibration detection mainly reflects the change in relative pose with time. From the aspects of detection based on the absolute pose and platform microvibration detection based on the relative pose, when the absolute pose obtained by a star sensor and an earth sensor is used for detection, the absolute pose obtained at the present discrete time often contains a large random error, and the accuracy is low when it is converted to the pose angular velocity (relative pose). When the relative pose obtained by the gyroscope and angular displacement sensor is used for detection, the high-frequency and continuous pose velocity is obtained. After eliminating the constant drift, the accuracy is high, and the detection sensitivity is obviously improved.

(3) In terms of universality, Table 3 shows that the implementation conditions are important factors in determining the microvibration detection method. The direct detection method based on the attitude sensor has no unfavorable factors under the implementation conditions, and the detection accuracy is high; thus, the universality is strong. In the indirect detection method based on a non-attitude sensor, the satellite image-based microvibration detection method has strong universality, the on-orbit satellites are basically equipped with multispectral or full-color sensors, and the current recording accuracy is high and easy to implement. The star-based microvibration detection method can be used as a supplementary means to monitor the microvibration of a satellite platform and has good universality. Based on the microvibration detection of surveying and mapping products, the universality is relatively poor because of the large error propagation. Microvibration detection based on laser altimeters is an emerging detection method. Because it needs laser altimeters to be equipped and the current measurement accuracy of these sensors is generally poor, the universality is relatively poor and requires improving the accuracy of subsequent measurements as a means of detection.

## 3. Experimental Analysis

In this paper, the ZY-3 satellite is used as the test object, and the platform microvibration is detected by using the ZY-3 satellite to cover the attitude measurement and non-attitude measurement of the load output data of the same area. The microvibration phenomenon existing in the ZY-3 satellite platform is comprehensively verified, and the microvibration law is comprehensively analyzed. Considering the significant representativeness of the platform microvibration in the initial stage of the satellite orbit, this paper selects the multispectral image, star map image, and star-sensitive gyro data of the ZY-3-01 000,381 track acquired at the same time on February 3, 2012, using various data and analyzes the change in microvibrations with time by using various data.

### 3.1. Direct Detection Method Based on a Non-Attitude Sensor

#### 3.1.1. Microvibration Detection Based on a Multispectral Image

**Figure 6:**
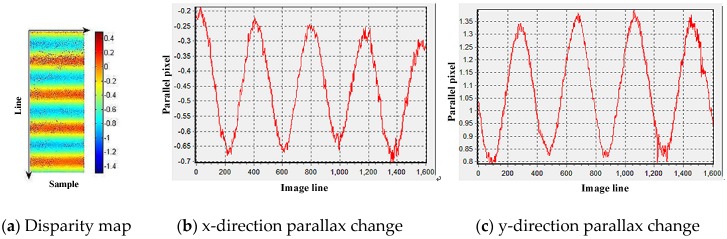
Multispectral image data analysis results.

Experiments were carried out on multispectral images, namely, the orbital 000,381 scenes 6-152, which have been preprocessed only by radiometric correction. For spectra one and two, a phase correlation algorithm was used for matching to obtain the spectral error map and error curve. Figure 6 reflects the changes in the multispectral camera image caused by microvibration. Through the Fourier transform, the vibration frequencies in the x and y directions are 0.642 Hz and 0.636 Hz, respectively, and the relative parallax amplitudes are 0.245 pixel and 0.251 pixel, respectively. The absolute attitude change of the satellite platform is obtained by absolute conversion of the relative parallax, and the absolute vibration amplitudes in the x and y directions are 0.505 pixel and 0.522 pixel, respectively.

#### 3.1.2. Star-Based Microvibration Detection

**Figure 7:**
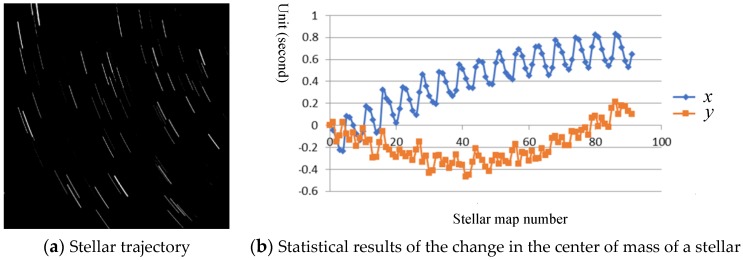
Stellar map data analysis results.

To facilitate the comparison, the experiment selected the ZY-3-01 orbit 000,381 stellar map, which was captured at the same time, to analyze the microvibration of the satellite platform. Figure 7a shows the stellar motion trajectory of the 000,381 orbital stellar map. The centroid extraction and trajectory fitting are performed on the stellar image points of the sequence star map. Figure 7b shows the statistical result of the difference between the statistical centroid coordinates and the centroid fit. In Figure 7, the residual of the microvibration detection based on the star map has a certain correlation with the residual in the row direction, mainly related to the centroid extraction algorithm and the moving speed of the star in the column direction. After analysis of the residuals in the row and column directions, the microvibration frequencies of the ZY-3 satellite in the rail/vertical rail direction are 0.51 Hz and 0.65 Hz, respectively.

### 3.2. Direct Detection Method Based on an Attitude Sensor

#### 3.2.1. Microvibration Detection Based on the Star Sensor Attitude

**Figure 8:**
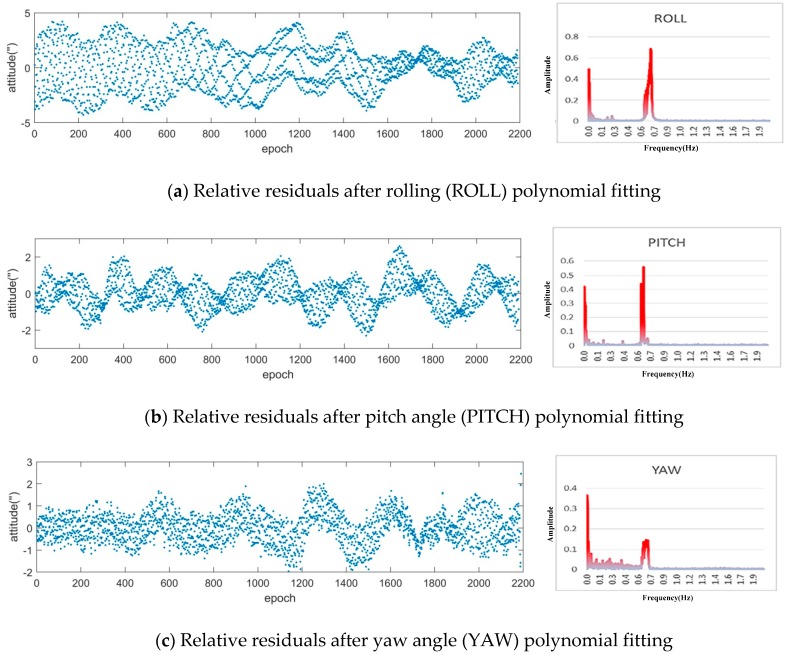
Three-axis attitude residual diagram after polynomial fitting (the left part is waveform and the right part is frequency).

The test uses the ZY-3 satellite 000,381 orbital sensor attitude data (sampling frequency of 4 Hz) and selects the 8th-order polynomial to fit the roll, pitch, and yaw attitude data. The variation in the three-axis attitude fitting residual is shown in Figure 8. The time domain is transformed into the frequency domain by the fast Fourier transform, and the frequency and vibration amplitude of the satellite attitude data are obtained. As shown in Figure 8, the ZY-3 satellite platform has a frequency range of 0.0–0.05 Hz and a 0.64–0.71 Hz microvibration frequency.

#### 3.2.2. Gyro-Based Microvibration Detection

**Figure 9:**
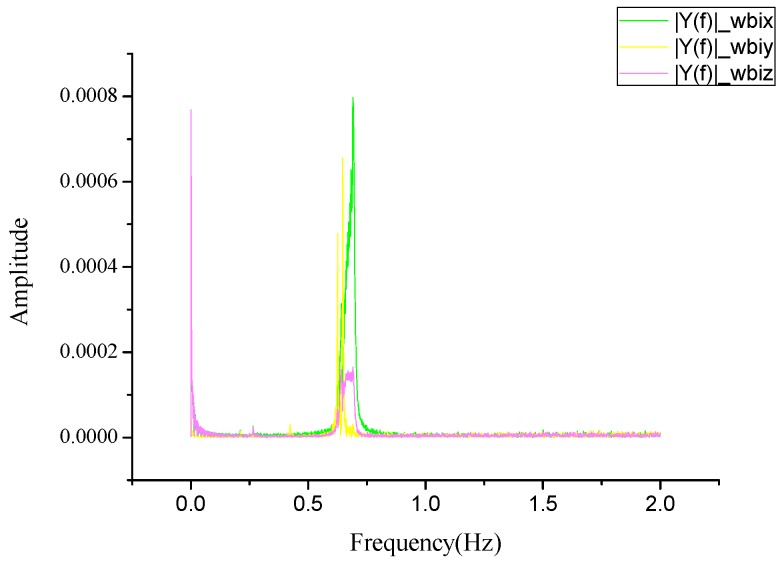
Analysis results of gyro data.

The test uses the ZY-3 satellite 000,381 rail gyro triaxial angular velocity data and directly analyzes the original three sets of gyro data by a Fourier transform. The experimental results are shown in Figure 9, which reveals that the frequency range of the ZY-3 satellite platform microvibration is approximately 0.0–0.05 Hz and 0.6–0.7 Hz.

#### 3.2.3. Microvibration Detection Based on the Angle of the Optical axis of the Multistar Sensor

**Figure 10:**
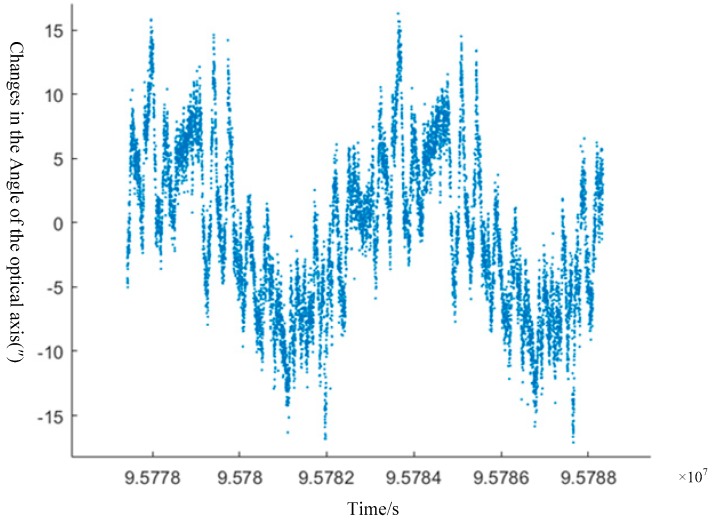
The variation in the angle of the star-sensitive optical axis.

The experiment uses the data of the 000,381 orbit original star sensor (A/B/C) transmitted by ZY-3-01 and selects the star-sensitive B+C to calculate the change in angle of the star sensor’s optical axis, as shown in Figure 10. Using the Fourier transform to analyze the frequency of change, the satellite platform microvibration is shown to have a frequency of approximately 0.0011 Hz.

The existing onboard load resources of the ZY-3 satellite are fully utilized, the multispectral image, star map, gyro data, star sensor attitude, and other data of the same period are selected, and the experimental results of five kinds of microvibration detection methods are given. The accuracies of the different detection methods are given under a constant weight, structure, size, composition, and track height, as shown in Table 5.

(1) Regarding the consistency of the detection frequency, the first four detection methods can detect the short-period microvibration phenomenon with a frequency of approximately 0.6~0.7 Hz in platform 3, and the detection results are consistent. At the same time, mutual verification can prove the reliability and accuracy of the detection method. The detection method based on the angle of the multi-star-sensitive optical axis can accurately detect the long-period microvibration of the satellite platform, which has a frequency of approximately 0.0011 Hz, and verify the correctness of the microvibration in the range of 0~0.05 Hz detected based on the attitude of the star sensor and the gyro. The generation of the long-period frequency is due to the influence of the temperature change over the orbit period during the operation of the satellite. Among these results, the detected microvibration frequency based on the star attitude is relatively low because of the inherent sampling frequency of the star sensor; thus, the detection frequency range is relatively limited.

(2) The accuracy of microvibration detection based on multispectral images depends on the matching accuracy, which can reach 0.02 pixels at present. Therefore, this method has obvious advantages in terms of reliability and accuracy, and it is the main technical means used in the short-period microvibration detection of the ZY-3 satellite. The precision of microvibration detection based on a star map depends on the precision of the star centroid extraction. At present, the centroid extraction precision can reach 0.1 pixel. This method can monitor the attitude change of the satellite platform all day and can be used as a supplementary means of detection in the actual production process. Based on the high detection accuracy of the angle change of the star sensor, long-period satellite platform microvibration detection can be realized without any interference from the meteorological conditions; this is the main means of long-period attitude detection of the ZY-3 satellite platform at present.

## 4. Discussion

The frequency of detection methods based on a non-attitude tracker is closely related to the sampling interval of the reference data. In terms of the optical image, star image, laser, and other auxiliary load data, this idea can be viewed in two different ways. One is that a single observation device can observe the change in a single target with time at a discrete time, such as a star point observed by a stellar camera or a beam of light emitted by a laser, and detect the microvibration of the platform through comparison with other references. Therefore, the detection frequency is related to the sampling frequency of the load itself, and the sampling frequency of this load is generally not high; thus, the detection frequency is often limited. The other is that two or more observation devices continuously observe multiple targets, such as the two or more spectral segments observed by a multispectral camera, and the relative amount of the same target observed by multiple observation devices changes with time. For detection, the sampling time is greatly shortened to the line scanning time interval; thus, the range of the platform microvibration detection frequency of these methods is greatly increased.

Based on the non-pose sensor detection method, the detection accuracy mainly depends on the quality of auxiliary load data and the processing accuracy of corresponding data, such as the image matching accuracy, star centroid extraction accuracy, laser spot centroid extraction accuracy, and so on. In contrast, the sensitivity when monitoring the same target change based on multiple pieces of observation equipment is better than that based on a single piece of observation equipment. For example, because of the short spectral interval, consistent time and imaging angle, the image registration accuracy is very high, reaching 1/50 pixel or even better, which directly reflects the parallax change caused by platform microvibration. When the stellar image points obtained by the stellar camera are detected, the 1/10 pixel centroid extraction accuracy reflects only the position at the current time but needs to accumulate a period of data fitting, reflect the flat vibration through the residual, and introduce the fitting error, which directly affects the detection sensitivity. The detection sensitivity based on the reference data is better than that based on the extended product. For example, the detection based on the laser footprint image spot needs the image square coordinates to be analyzed, while the detection based on the footprint coordinates needs the image square coordinates to be converted to the object side and various errors such as those of the pose orbit, calibration, etc., and that related to the accuracy of the ground reference data to be introduced, which greatly reduces the detection sensitivity.

In general, from the detection sensitivity and detection frequency range, in the indirect detection method with a non-attitude sensor, that is, the satellite-image-based microvibration detection method, where the same phase data are obtained by parallel observation, the matching precision is high. This method is currently the mainstream means of detection and is also a hot topic of current research. In the direct detection method with the attitude sensor, the detection method based on a high-frequency angular velocity sensor such as an ADS is also recommended. Not only is this method carried out in earth observation satellites such as those of Japan, but also the relevant load is carried on high-resolution remote sensing satellites of China. Considering that the optical image acquisition is affected by the weather, the direct detection method based on the attitude sensor and the indirect detection method based on the star map are more suitable for long-term on-orbit monitoring. The methods based on optics and lasers can be used as supplements for the dynamic monitoring of key regions around the world.

## 5. Conclusions

Satellite platform microvibration is an inevitable phenomenon during orbital operation, and is an important challenge for high-precision processing of high-resolution optical satellites. Based on research at home and abroad, the data transmitted from the ZY-3 platform are used in this paper. We found that the ZY-3 platform has a short-period frequency of about 0.6 to 0.7 Hz and a long-period frequency of 0.0011 Hz. In the non-attitude sensor detection method, the microvibration detection method based on satellite image has high accuracy, it is the mainstream detection method at present, and it is also the current research hotspot. In the attitude sensor detection method, the detection method based on ADS will be the main technical method in the future. This paper comprehensively analyzes the microvibration detection methods of satellite platforms in order to provide theoretical basis for subsequent microvibration research.

First, the non-attitude sensor detection method is an indirect detection method based on auxiliary load data, which is suitable for detecting satellite platform microvibrations in a short time. The detection accuracy is mainly affected by the quality of the auxiliary data and processing accuracy. With continuous improvement of the auxiliary load, the detection accuracy and detection range will also be improved. Multispectral image data and stellar map image data are used for microvibration detection in the experiment, and the two methods are mutually examined to verify the existence of short-period microvibrations in the ZY-3 platform, with a frequency range of 0.6~0.7 Hz.

Second, the attitude sensor detection method is a direct detection method that uses attitude sensors as a reference and is suitable for the long-term detection of satellite platform microvibrations. It is not restricted by the meteorological conditions, and the detection accuracy is mainly affected by the measurement accuracy and frequency of the attitude sensor itself. The attitude data captured by the track sensor, gyroscope data, and multiple satellite-sensitive optical axis angles are collected in the experiment. The track sensor with gyro attitude data detects a frequency range of 0.6~0.7 Hz, and a frequency range of 0~0.05 Hz is detected for long-period microvibrations. Based on the star-sensor-angle detection of the long-period microvibration frequency, the frequency is mainly concentrated at 0.0011 Hz. Various methods of mutual authentication are used to determine the existence of the long-period microvibration frequency. Compared with the short-period amplitude, the long-period amplitude is relatively large, which has a more significant impact on the geometric positioning accuracy. The subsequent compensation of the long-period microvibration can offer assurance regarding the mapping satellite data used in 1:50,000 global uncontrollably surveyed maps.

In the future, satellite sensors will be transformed to acquire very-high-spatial-resolution data, and the agility of the satellite platform will be improved. The influence of microvibrations on the geometric accuracy and mapping accuracy of satellite images will become increasingly significant. Using these methods comprehensively, an integrated microvibration detection method can be developed in the future.

## Figures and Tables

**Table 1:** Microvibration detection results for satellite platforms at home and abroad.

Satellite/Sensor	Year	Spectral Type	Spatial Resolution (m)	Frequency ^a^ (Hz)	Amplitude ^b^ (m)
ASTER [9,10,11]	1999	Near-infrared/short-wave infrared	15/30	1.5–1.6	6–7
QuickBird [10]	2001	Panchromatic/Multispectral	0.61/2.44	1, 4.3	2.5, 0.1
SPOT 5 [10]	2002	Panchromatic/Multispectral	2.5/10	approximately 0.003	approximately 20
ALSat-1 [12]	2002	Multispectral	32	0.5	-
Nigeria Sat [12]	2003	Panchromatic/Multispectral	2.5/5	0.5	-
UK-DMC [12]	2003	Multispectral	22	0.6	-
MEX-HRSC [13,14]	2003	Panchromatic	10	0.1-0.2,1.7	8
Beijing-1 [15]	2005	Panchromatic/Multispectral	4/32	200	3
ALOS [16,17]	2006	Panchromatic/Multispectral	2.5/10	under 6	-
Kompsat-2 [18]	2006	Panchromatic/Multispectral	1.0/4	210	0.14
Mapping Satellite-1 [19]	2010	Panchromatic/Multispectral	2/10	0.105, 0.635, 4	0.2, 0.1, 0.1
Pleiades-HR [20]	2011	Panchromatic/Multispectral	0.5/2.0	70.9–78.4	0.14
ZY3-01 [21,22,23,24,25,26]	2012	Panchromatic/Multispectral	2/5	0.6-0.7	2.5-7.5

^a^ Satellite attitude jitter frequency may be detected in roll, pitch, or both; the frequencies listed here are from either or both. ^b^ The amplitude has been transformed from onboard arcsecs or pixels to ground meters (from satellite to ground), intuitively revealing the influence of jitter. A lack of amplitudes listed in the existing records and research is indicated by “-”.

**Table 2:** Detection frequency range comparison.

Category	Detection Method	Influence Factors of Detection Frequency Range	Theoretical Detection Frequency Range/Hz
Indirect detection method based on a non-attitude tracker [45,46,47,48,49,50]	Microvibration detection based on satellite imagery	Sampling interval of image scan line	Line frequency level
	Microvibration detection based on a star image	Sampling interval of star image	Less than one-half the sampling frequency of the star image
	Microvibration detection based on laser footprint coordinate changes	Repeat sampling frequency of laser spot	Less than one-half the laser repeat frequency
	Microvibration detection based on the laser footprint image point	Sampling interval of footprint image	Less than one-half the sampling frequency of the footprint image
	Microvibration detection based on surveying products	Line sampling interval of image	Line frequency level
Direct detection method based on an attitude tracker [35,51]	Microvibration detection based on the star tracker attitude	Sampling frequency of star tracker	Less than one-half the sampling frequency of the star tracker
	Microvibration detection based on an angular velocity tracker	Sampling frequency of angular velocity tracker	Less than one-half the sampling frequency of the angular velocity sampling frequency

**Table 3:** Detection sensitivity comparison.

Category	Detection Method	Influence Factors of Detection Sensitivity	Detection Sensitivity (Taking the Current Mainstream Tracker as an Example)
Indirect detection method based on a non-attitude tracker [21,22,23,24,25,26,52,53,54]	Microvibration detection based on satellite imagery	Image registration accuracy	0.01~0.05 pixel
	Microvibration detection based on a star image	Stellar centroid extraction accuracy	0.05-0.1 pixel
	Microvibration detection based on laser footprint coordinate changes	Laser calibration accuracy, attitude and track accuracy, laser pointing accuracy and mounting stability	2~5″
	Microvibration detection based on the laser footprint image point	Spot centroid extraction accuracy	0.05-0.1 pixel
	Microvibration detection based on surveying products	Matching accuracy, surveying product accuracy	0.05~0.1 pixel
Direct detection method based on an attitude tracker [33,34,35]	Microvibration detection based on the star tracker attitude	Star tracker measurement accuracy	1~2″
	Microvibration detection based on an angular velocity tracker	Angular velocity tracker measurement accuracy	0.01-0.1″

**Table 4:** Comparison of universality.

Category	Detection Method	Implementation Conditions	Universality
Indirect detection method based on a non-attitude tracker [21,22,23,24,25,26,52,53,54]	Microvibration detection based on satellite imagery	Optical multispectral, panchromatic image data	Highly precise and easy to implement, but data acquisition is susceptible to weather.
	Microvibration detection based on a star image	Downstream long-term original star image	Fast processing speed, data are not affected by the weather, and it can be monitored for a long time.
	Microvibration detection based on laser footprint coordinate changes	Dependent on ground reference data	The calculation is simple and easy to analyze, but the error propagation is large and the universality is relatively poor.
	Microvibration detection based on the laser footprint image point	Equipped with a monitoring-laser-footprint-related load such as a print camera, optical axis monitoring camera, etc.	The processing speed is fast, it is easy to analyze, but the universality is poor.
	Microvibration detection based on surveying products	Depending on the accuracy of auxiliary information such as the ground reference, the long-time-series cloud-free image products can be difficult to obtain	Affected by the external conditions, the universality is poor.
Direct detection method based on an attitude tracker [33,34,35]	Microvibration detection based on the star tracker attitude	N/A	Not affected by the external conditions, the calculation is simple, fast, easy to analyze, and very universal.
	Microvibration detection based on an angular velocity tracker	N/A	It is not affected by the external conditions, with high accuracy, a wide detection range and strong universality.

**Table 5:** Comparison of the results of five microvibration detection methods.

Detection Method	Experimental Data	Frequency/Hz	Amplitude/Angular Seconds	Sensitivity/Angular Seconds
Microvibration frequency detection based on satellite imagery	multispectral image	0.642 (*x* direction)/0.636 (*y* direction)	0.5–1	0.01
Star-based microvibration detection	star map	0.65 (*x* direction)/0.51 (*y* direction)	0.5–1	7
Microvibration detection based on the star sensor attitude	attitude data	0–0.05, 0.6–0.70	0.5–1	5
Gyro-based microvibration detection	attitude data	0–0.05, 0.64–0.71	0.5–1	2
Microvibration detection based on the angle of the optical sensor’s optical axis	attitude data	0.0011	7	1.8
